# Photoacclimatory Responses of *Zostera marina* in the Intertidal and Subtidal Zones

**DOI:** 10.1371/journal.pone.0156214

**Published:** 2016-05-26

**Authors:** Sang Rul Park, Sangil Kim, Young Kyun Kim, Chang-Keun Kang, Kun-Seop Lee

**Affiliations:** 1 Estuarine & Coastal Ecology Laboratory, Department of Marine Life Sciences, Jeju National University, Jeju, Republic of Korea; 2 Department of Biological Sciences, Pusan National University, Pusan, Republic of Korea; 3 School of Environmental Science & Engineering, Gwangju Institute of Science and Technology, Gwangju, Republic of Korea; University of California - Davis, UNITED STATES

## Abstract

Photoacclimatory responses of the seagrass *Zostera marina* in the intertidal and subtidal zones were investigated by measuring chlorophyll *a* fluorescence parameters, photosynthetic pigments, leaf δ^13^C values, and shoot morphology in two bay systems. Intertidal plants had higher carotenoid concentrations than subtidal plants to avoid photodamage under excess light conditions during the day. The maximum relative electron transport rate (rETR_max_) and minimum saturation irradiance (E_k_) of the intertidal plants were higher than those of the subtidal plants, whereas photosynthetic efficiency (α) and maximum quantum yield (*F*_*v*_/*F*_*m*_) were higher in subtidal plants. The intertidal plants also had significantly greater Stern–Volmer non-photochemical quenching (NPQ) than that of the subtidal plants. These results suggest that the subtidal plants photoacclimated to use limited light more efficiently, and the intertidal plants exhibited photosynthetic responses to minimize photodamage at excess irradiance. The δ^13^C values of leaf tissues were more negative in the intertidal plants than those in the subtidal plants, suggesting that the intertidal plants used atmospheric or dissolved CO_2_ for photosynthesis during emersion. Effective quantum yield (*ΔF*/*F*_*m*_*´*) in the intertidal plants decreased more slowly after emersion than that in the subtidal plants, indicating higher desiccation tolerance of the intertidal plants. The intertidal plants also recovered more rapidly from desiccation damage than the subtidal plants, suggesting photosynthetic adaptation to desiccation stress. The photosynthetic plasticity of *Z*. *marina* in response to variable environmental conditions most likely allows this species to occur in the intertidal and subtidal zones.

## Introduction

Seagrasses occurring in the intertidal and subtidal zones are exposed to highly variable environmental conditions due to tidal changes [1,2]. Seagrasses in the intertidal zone are regularly exposed to air and consequently experience extreme high and low temperatures, high photoinhibitory irradiance, and desiccation stress relative to subtidal seagrass [2–4]. Such extreme temperatures can lead to significant seagrass dieback when seagrasses are exposed to air during low tide [5–7]. Desiccation stress during low tide has been considered the primary factor limiting seagrass distribution at the upper intertidal zone [8]. Seagrasses residing the intertidal zone are usually smaller than those in the subtidal zone to minimize the effects of emergence stress [9,10]. Intertidal seagrasses also show light-dependent responses, such as decreased photosynthetic efficiency and increased photoprotection during periods of high irradiance and air exposure [11,12].

In contrast, seagrasses in the subtidal zone adapt to reduced light conditions caused by light attenuation and scattering due to the overlaying water column and suspended particles [13,14]. Seagrasses in the deep subtidal zone generally have longer leaves and wider leaf blades than those in the shallow subtidal or intertidal zone, which allows more photosynthesis, in turn resulting in greater growth [4]. Seagrasses also respond to reduced light conditions by increasing chlorophyll content and decreasing the chlorophyll *a/b* ratio to enhance light absorption efficiency by using the abundant wavelengths efficiently [15–17]. As seagrasses in the intertidal and subtidal zones are under highly different light conditions, they exhibit distinctly different photoacclimatory responses to maximize photosynthetic activity and photoprotection from excess irradiance.

Seagrasses assimilate large amounts of inorganic carbon to achieve high level production [18,19]. Marine macrophytes, including seagrass, use both CO_2_ and HCO_3_^−^ for photosynthetic carbon reduction [20–23]. Despite air exposure during low tide, seagrasses in the intertidal zone can continue to photosynthesize utilizing CO_2_ in the air [24]. Thus, the composition of inorganic carbon sources for seagrass photosynthesis probably varies between intertidal and subtidal plants. Because carbon stable isotope ratios of plant tissues change based on the inorganic carbon sources for photosynthesis [25,26], seagrasses in the intertidal and subtidal zones may have different carbon stable isotope ratio ranges.

Although eelgrass, *Zostera marina*, which is the most abundant seagrass species in the Northern Hemisphere, usually occurs in the shallow subtidal zone [27–29], intertidal *Z*. *marina* populations have also been reported from various geographical locations [2,30,31]. Despite their distribution in the intertidal and subtidal zones, the ecological and physiological differences between intertidal and subtidal *Z*. *marina* plants have rarely been studied within a bay system. Compared to morphological plasticity, photoacclimatory responses and inorganic carbon sources of intertidal and subtidal *Z*. *marina* plants are not well documented. Thus, in the present study, we examined the photoacclimatory responses of intertidal and subtidal *Z*. *marina* plants by measuring chlorophyll *a* fluorescence parameters. We also evaluated the variability in the inorganic carbon sources available to intertidal and subtidal plants by measuring carbon stable isotope ratios of *Z*. *marina* leaf tissues. Additionally, we conducted emersion experiments to compare desiccation tolerance and recovery ability from emersion stress between intertidal and subtidal *Z*. *marina*. We hypothesized that intertidal *Z*. *marina* would exhibit adaptive responses to an environment of harsh extremes, whereas subtidal plants would exhibit photoacclimatory responses to low irradiance. Carbon stable isotope ratios of intertidal plants are assumed to be more negative than those of subtidal plants because intertidal plants can utilize CO_2_ in the air during low tide. Global climate change-associated environmental disturbances, such as a rise in sea level, may lead to changes in tidal level, and consequently cause significant changes in the distribution and population structure of seagrasses in intertidal and subtidal zones. Thus, this study will provide valuable information for a better understanding of the effects of global climate change on coastal seagrass ecosystems.

## Materials and Methods

### Study sites

**Fig 1:**
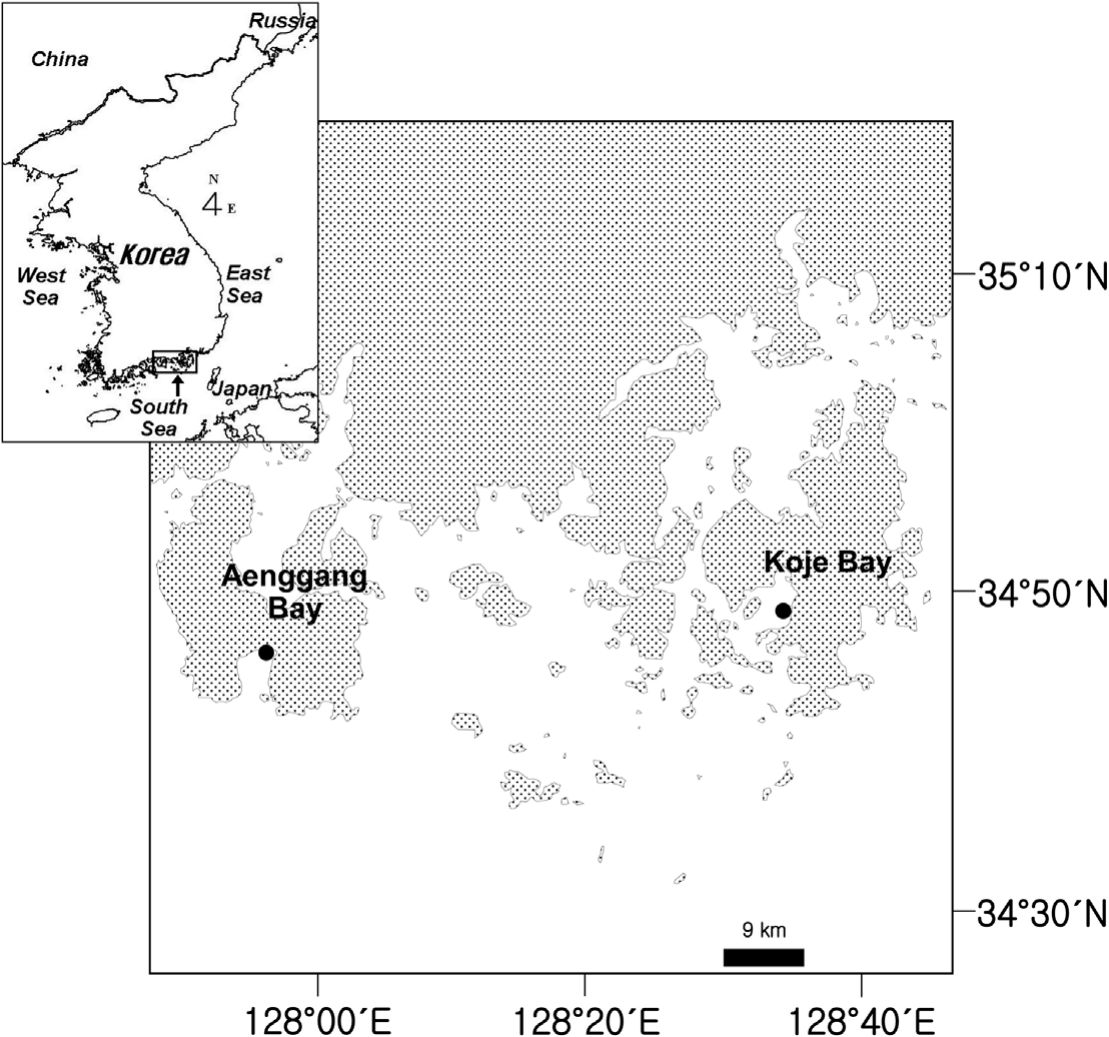
Study sites in Aenggang Bay and Koje Bay on the southern coast of the Korean peninsula.

The study sites were located in Aenggang Bay (34° 46΄N, 127° 56΄E) and Koje Bay (34°48΄N, 128°35΄E) on the southern coast of Korea (Fig 1), where four *Zostera* species (*Z*. *japonica*, *Z*. *marina*, *Z*. *caespitosa*, and *Z*. *caulescens*) are distributed at different water depths. No specific permissions to collect research samples were required at the study sites in Aenggang Bay and Koje Bay, and the field study did not involve endangered or protected species. This study was conducted in monotypic meadows of *Z*. *marina*, which is the dominant species in both bay systems. *Zostera marina* was distributed continuously from the lower intertidal to the upper subtidal zones at both study sites, and thus plants at the intertidal and subtidal zones were from the same population. The plants in the lower intertidal zone were exposed to air during low tide. The emersion period for the intertidal plants ranged from 0.8 hours per day in July-September and 3.4 hours per day in March, with a mean emersion period of 1.7 hours per day. In contrast, the subtidal plants were never exposed to air in either bay system and were located at a mean water depth of about 3.0 m relative to mean lower low water. In Koje Bay, average shoot density (1140 shoots m^-2^) in the intertidal zone was 11.6 times higher than that (98 shoots m^-2^) in the subtidal zone [32,33]. Average daily underwater irradiances were 22.1 and 11.7 μmol photons m^-2^ d^-1^ in the intertidal and subtidal zones, respectively, in Koje Bay [32,33]. Water temperatures were 5.0–28.5°C, and salinity was 30.4–33.6. Sandy sediments predominated at both study sites. Water column NH_4_^+^ and PO_4_^3−^ concentrations were usually less than 4 μM and 2 μM, respectively; mean NO_3_^−^ + NO_2_^−^ concentration in the water column was about 1.7 μM [34]. The tidal amplitude at both sites varied between 4.0 m during spring tide to 1.2 m during neap tide, and the tidal regime is semi-diurnal with an approximate 3.0 m tidal range (Tide tables for Coast of Korea, National Oceanographic Research Institute of Korea).

### Biological measurements

Ten to fifteen mature terminal shoots were collected from each intertidal and subtidal site in the two bay systems on a seasonal basis (April, July, and October 2006 and February 2007) to measure shoot morphological characteristics. Sheath length was measured from the meristem to the top of the sheath. Shoot height was measured from the meristem to the tip of the longest leaf to the nearest 1.0 mm, and the width of the longest leaf was measured to the nearest 0.1 mm.

Concentrations of leaf pigments (chlorophylls and carotenoids) were estimated using the middle part of the youngest mature (fully grown) leaf where the chlorophyll *a* fluorescence parameters were measured. Six replicate samples from each site were collected and cleaned of epiphytes in the laboratory. Pre-weighed leaf tissues were extracted with 5.0 ml of N,N-dimethylformamide for 2–3 days in glass tubes following the method of Dunton and Tomasko [35]. Absorbance of the extracts was measured at 480, 647, 664, and 750 nm using a Shimadzu Model 2264 UV spectrophotometer (Tokyo, Japan). Pigment concentrations were calculated using the extinction coefficient equations [36].

### Measurement of the chlorophyll *a* fluorescence parameters

The chlorophyll *a* fluorescence parameters were measured *in situ* using 10–15 shoots from each site. The parameters were measured at the middle part of the youngest mature leaf using a pulse amplitude modulated (PAM) fluorometer (Diving-PAM; Heinz Walz GmbH, Effeltrich, Germany) with an 8 mm fiber optic. Before measuring rapid-light curves (RLCs), maximum quantum yield (*F*_*v*_/*F*_*m*_) of photosystem II (PSII) was determined after a 10 min dark adaptation using the following equation: *F*_*v*_/*F*_*m*_ = (*F*_*m*_−*F*_*0*_) / *F*_*m*_, where *F*_*0*_ is the minimal fluorescence of a dark-adapted leaf, in which all PSII reaction centers are open, and *F*_*m*_ is the corresponding maximum fluorescence measured with all PSII reaction centers closed following a short (0.8 s) saturating light period (e.g., [37]). Effective quantum yield of PSII (*ΔF*/*F*_*m*_*´*) was measured on light-adapted samples according to the relationship: *ΔF*/*F*_*m*_*´* = (*F*_*m*_*´* –*F*_*t*_)/*F*_*m*_*´*, where *F*_*m*_*´* is the light-adapted maximum fluorescence and *F*_*t*_ is the fluorescence before a saturating pulse [38].

RLCs were produced by the Diving-PAM under control of an internal program using artificial photosynthetic photon flux density (PPFD). The samples were exposed to eight incremental steps of irradiance ranging from 0 to 1,042 μmol photons m^−2^ s^−1^. The samples were exposed to 10 s of irradiance at each incremental step during the RLC measurement. The relative electron transport rate (rETR) was determined from the following equation: rETR = *ΔF/F*_*m*_*´* × PPFD × 0.5 × 0.84, where *ΔF*/*F*_*m*_*´* is the effective quantum yield of PSII, 0.84 is the assumed absorption coefficient, and 0.5 is a correction for the two photosystems absorbing photons [38]. The RLC data were fitted to the model of Platt et al. [39] to obtain values of photosynthetic efficiency (*α*), minimum saturating irradiance (E_k_), and maximum relative electron transport rate (rETR_max_). E_k_ was calculated as E_k_ = rETR_max_/*α*. Quenching coefficients were calculated according to the following equations [40].

Photochemical quenching $\left(qP)=(F_{m}^{´}-F)/(F_{m}^{´}-F_{0}\right)$

Non-photochemical quenching $\left(qN)=({F_{m}-F}_{m}^{´})/(F_{m}-F_{0}\right)$

Stern–Volmer non-photochemical quenching $(NPQ)=({F_{m}-F}_{m}^{´})/F_{m}^{´}$

### Stable isotope analysis

We used the youngest mature leaf (second or third leaf) of the shoots collected for morphological characteristic to determine the carbon stable isotope ratio (δ^13^C) of leaf tissue. Leaf samples were dried at 60°C to a constant weight and then ground using a mortar and pestle. The ground samples were wrapped in tin capsules and combusted at high temperature (1030°C) in an elemental analyzer (Eurovector 3000 Series; Milan, Italy), and the CO_2_ gas was analyzed to determine the carbon stable isotope ratio using a continuous flow-through inlet system attached to an isotope ratio mass spectrometer (Isoprime; GV Instruments, Manchester, UK). Peedee belemnite marine limestone was used as the primary standard. Analytical precision was approximately 0.1–0.2‰ for δ^13^C.

### Photosynthetic responses to emersion and recovery after re-immersion

To compare the photosynthetic responses to emersion between intertidal and subtidal *Z*. *marina* plants, we collected 10 shoots from each tidal zone in the two bay systems. The plants were maintained in 20°C aerated seawater with underwater irradiance of 150 μmol photons m^−2^ s^−1^ in the laboratory. All emersion experiments were conducted within 4 h of shoot collection. The plants were placed under air-exposed conditions within an environmentally controlled room with constant temperature (20°C), relative humidity (65%), and light intensity (300 μmol photons m^−2^ s^−1^). Effective quantum yield was measured every hour (0, 1, 2, 3, 4, 5, and 6 h) after air exposure.

*Zostera marina* shoots were exposed to air for 1, 2, 3, 4, 5, and 6 h periods and immediately re-immersed into seawater with constant temperature (20°C) and underwater irradiance (150 μmol photons m^−2^ s^−1^) to investigate recovery of photosynthetic efficiency from emersion stress. The effective quantum yields of the shoots were measured 0, 1, 3, 5, and 12 h after re-immersion.

### Statistical analyses

All values are presented as mean ± standard error. Data were log (*x* + 1) or arcsin (x/1000 + 1) transformed to meet the assumptions of parametric statistics prior to analysis. Significant differences in morphological characteristics (shoot height, sheath length, and leaf width), RLC parameters (rETR_max_, α, E_k_, maximum quantum yield, and NPQ), photosynthetic pigments (total chlorophyll, total carotenoids, chlorophyll *a*/*b* ratio, and the total chlorophyll/total carotenoid ratio), and the carbon stable isotope ratio (δ^13^C) between the intertidal and subtidal plants and among seasons in each bay system were analyzed using a two-way analysis of variance (ANOVA). Significant differences in effective quantum yield between the intertidal and subtidal plants and among air exposure periods were also tested using a two-way ANOVA. When a significant difference was observed among variables, the means were analyzed using the Student–Newman–Keuls test to determine where the significant difference occurred. An alpha level of 0.05 was used for all statistical tests. All statistical analyses were performed using SPSS ver. 18.0 (SPSS Inc., Chicago, IL, USA).

## Results

### Shoot morphology and photosynthetic pigments

Shoot height, sheath length, and leaf width of *Z*. *marina* in the intertidal zone were significantly shorter or narrower (*P* < 0.001 for all) than those in the subtidal zone throughout the experimental period in both bay systems (Table 1; S1 Table). The greatest differences in shoot morphology between the intertidal and subtidal plants occurred during spring–summer periods when the subtidal populations exhibited their highest growth rates (Table 1).

**Table 1:** Morphological characteristics (shoot height, sheath length and leaf width) of intertidal and subtidal *Zostera marina* plants in Aenggang Bay and Koje Bay.

		Aenggang Bay		Koje Bay	
Season	Morphological characteristic	Intertidal	Subtidal	Intertidal	Subtidal
**Spring**	Shoot height (cm)	34.5 ± 2.1^a^	136.5 ± 6.3^b^	37.2 ± 1.8^a^	82.0 ± 4.4^b^
	Sheath length (cm)	7.4 ± 0.4^a^	32.2 ± 1.1^b^	9.9 ± 0.6^a^	19.9 ± 1.0^b^
	Leaf width (mm)	6.2 ± 0.3^a^	11.6 ± 0.3^b^	5.7 ± 0.2^a^	9.9 ± 0.3^b^
**Summer**	Shoot height	24.8 ± 1.1^a^	119.1 ± 3.8 ^b^	47.4 ± 3.3^a^	98.8 ± 2.8^b^
	Sheath length	5.0 ± 0.2^a^	15.2 ± 1.0^b^	9.3 ± 0.4^a^	23.3 ± 0.6^b^
	Leaf width	4.6 ± 0.2^a^	9.5 ± 0.1^b^	6.9 ± 0.2^a^	9.6 ± 0.2^b^
**Fall**	Shoot height	36.0 ± 2.8^a^	51.2 ± 1.6^b^	36.5 ± 2.0^a^	65.0 ± 1.4^b^
	Sheath length	7.4 ± 0.7^a^	10.6 ± 0.3^b^	8.0 ± 0.5^a^	13.9 ± 0.2^b^
	Leaf width	5.8 ± 0.2^a^	8.2 ± 0.2^b^	6.1 ± 0.2^a^	8.2 ± 0.3^b^
**Winter**	Shoot height	41.1 ± 2.2^a^	55.0 ± 1.5^b^	32.7 ±1.8^a^	55.2 ± 1.4^b^
	Sheath length	8.0 ± 0.3^a^	14.6 ± 0.8^b^	8.3 ± 0.8^a^	16.4 ± 0.1^b^
	Leaf width	6.2 ± 0.3^a^	9.5 ± 0.2^b^	5.2 ± 0.2^a^	10.0 ± 0.4^b^

Values are mean ± standard errors (n = 10–15). Values with the same letter are not significantly different (*P* < 0.05) between the intertidal and subtidal plants in each bay system and each season.

**Fig 2:**
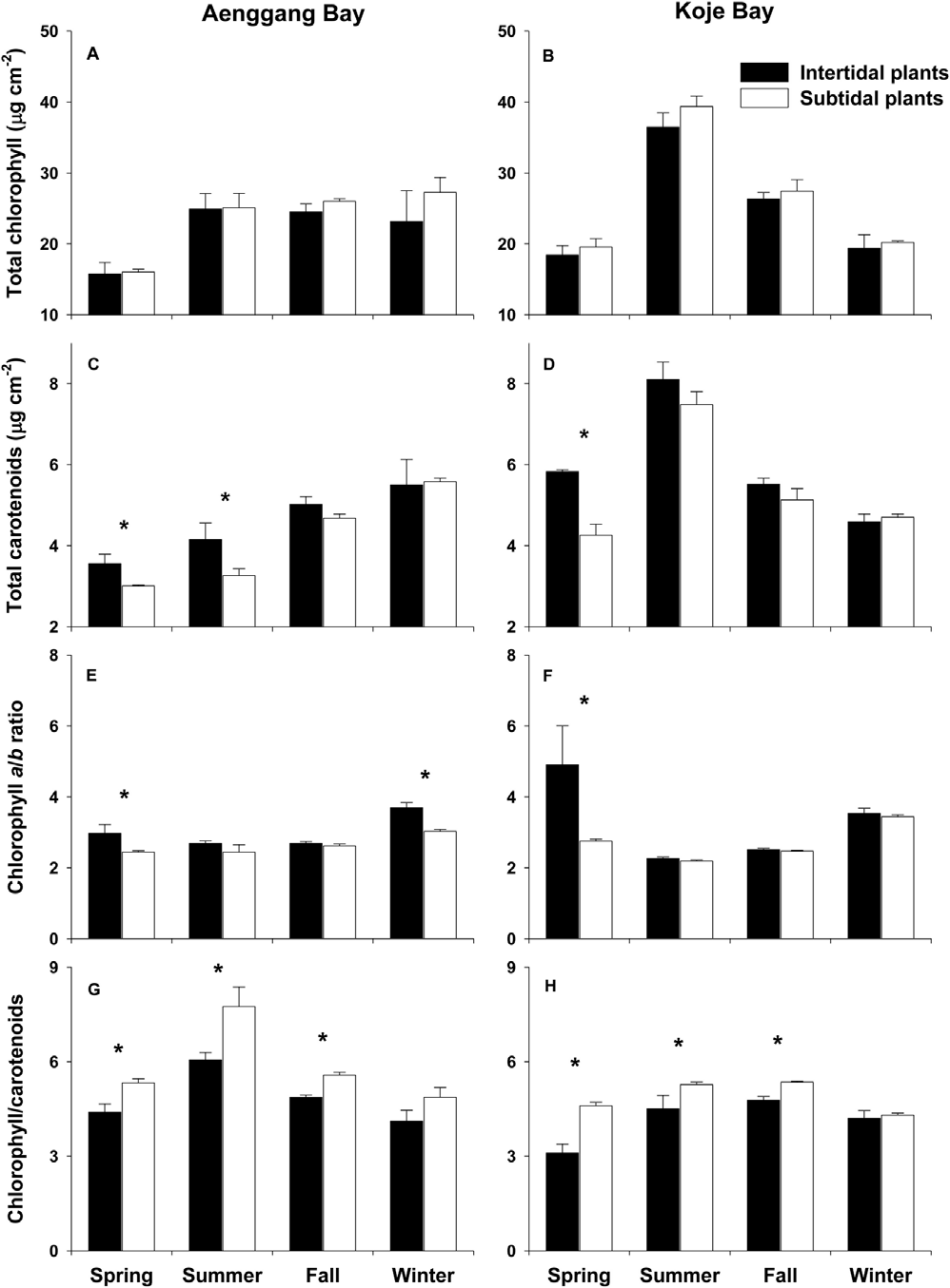
*Zostera marina*. **Photosynthetic pigment concentrations (total chlorophyll and total carotenoids), chlorophyll *a*/*b* ratio, and the chlorophyll/carotenoid ratio of the intertidal and subtidal plants in Aenggang Bay (A, C, E, and G) and Koje Bay (B, D, F, and H).** Values are means ± standard errors (n = 6). An asterisk above the bar indicates a significant difference between the intertidal and subtidal plants in each season.

Total chlorophyll (chl. *a* + chl. *b*) content was slightly higher in the subtidal plants than in the intertidal plants, but was not significantly (*P* = 0.226 in Aenggang Bay; *P* = 0.174 in Koje Bay) different between the intertidal and subtidal plants (Fig 2A and 2B; S1 Table). Total carotenoid concentrations were significantly higher (*P* < 0.05 in Aenggang Bay; *P* < 0.01 in Koje Bay) in the intertidal plants than in the subtidal plants during spring and summer in Aenggang Bay and during spring in Koje Bay (Fig 2C and 2D; S1 Table). Chlorophyll *a*/*b* ratios of the intertidal plants were significantly higher (*P* < 0.01 in Aenggang Bay; *P* < 0.05 in Koje Bay; S1 Table) than those of the subtidal plants during spring and winter in Aenggang Bay and during spring in Koje Bay (Fig 2E and 2F; S1 Table). In contrast, the chlorophyll/carotenoid ratios were significantly higher (*P* < 0.05 in Aenggang Bay; *P* < 0.001 in Koje Bay) in the subtidal plants than in the intertidal plants during spring–fall in both bay systems (Fig 2G and 2H; S1 Table).

### Chlorophyll *a* fluorescence parameters

**Fig 3:**
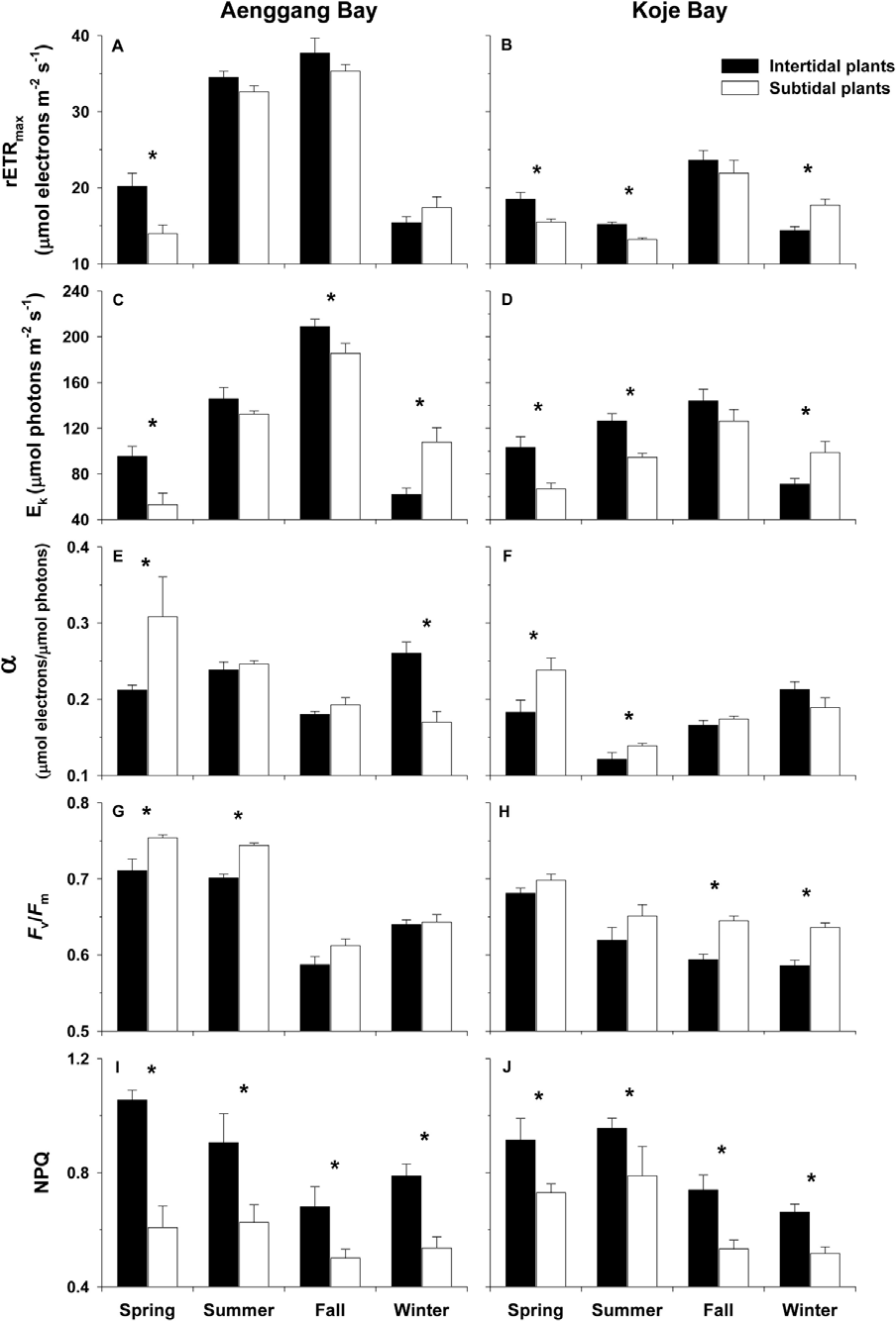
*Zostera marina*. **Parameters (rETR**_**max**_**, α, E**_**k**_**, *F***_***v***_**/*F***_***m***_**, and NPQ) derived from the rapid light curves (RLC) of intertidal and subtidal plants in Aenggang Bay (A, C, E, G, and I) and Koje Bay (B, D, F, H, and J).** Stern–Volmer non-photochemical quenching (NPQ) values were obtained at the highest actinic irradiance. Values are means ± standard errors (n = 10–15). An asterisk above the bar indicates a significant difference between the intertidal and subtidal plants in each season.

The rETR_max_ and E_k_ values of the intertidal plants tended to be higher than those of the subtidal plants during all experimental periods, except winter (Fig 3A–3D; S2 Table). The α values exhibited the opposite trend to that of rETR_max_ and E_k_ (Fig 3E and 3F; S2 Table). The α values were higher in subtidal plants than in intertidal plants during spring–fall. Subtidal plant *F*_*v*_/*F*_*m*_ values were significantly higher (*P* < 0.001 in both bay systems) than those of the intertidal plants in both bay systems (Fig 3G and 3H; S2 Table). The intertidal plants had significantly greater (*P* < 0.001) NPQ than that of the subtidal plants in both bay systems throughout the experimental period (Fig 3I and 3J; S2 Table). Mean NPQ values (0.83 and 0.76 in Aenggang Bay and Koje Bay, respectively) in intertidal plants were 1.3–1.5 times higher than in subtidal plants (0.56 and 0.59).

**Fig 4:**
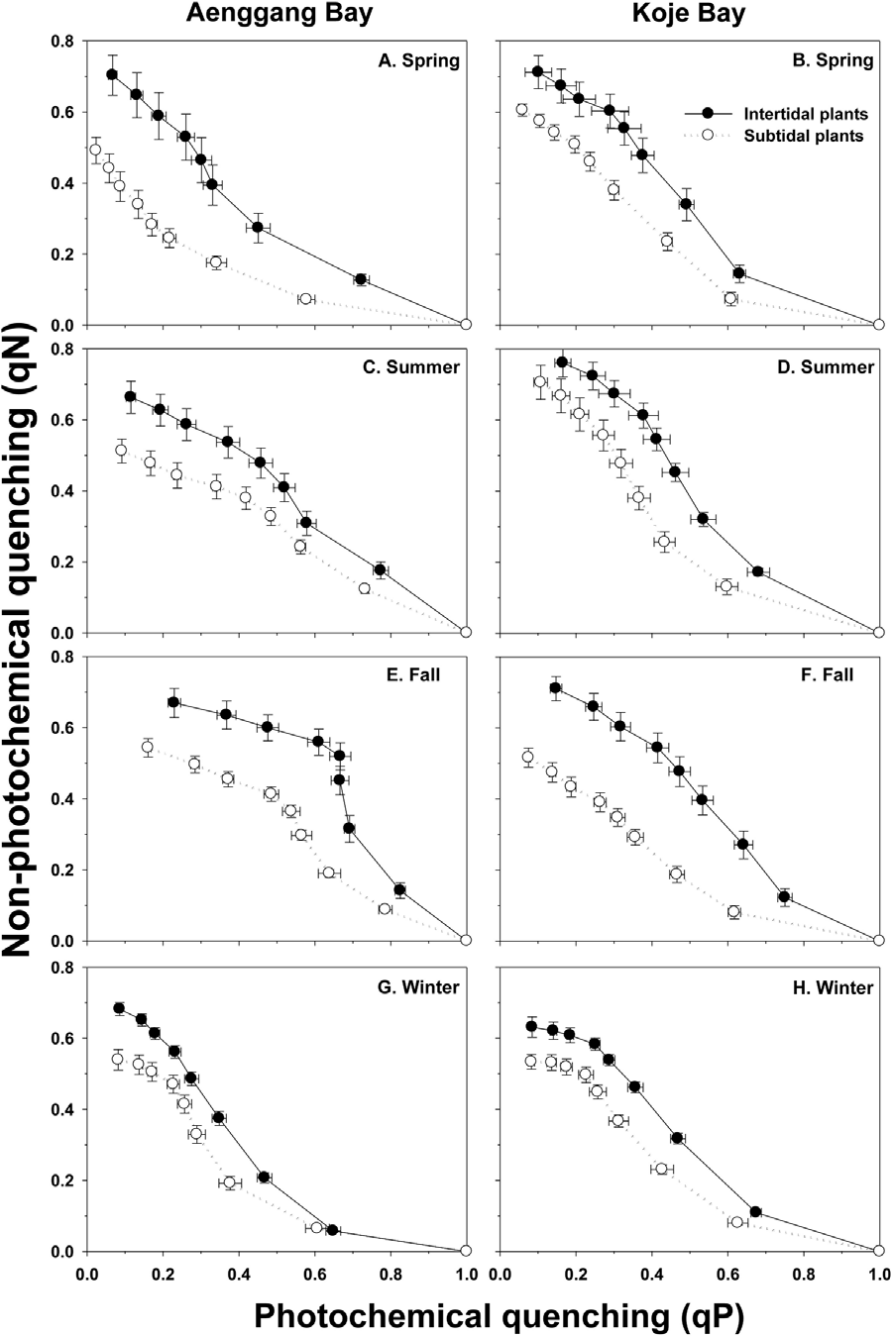
*Zostera marina*. **Relationship between photochemical quenching (qP) and non-photochemical quenching (qN) during each season in Aenggang Bay (A, C, E, and G) and Koje Bay (B, D, F, and H).** The rapid light curve (RLC) measurements were taken from the lowest to highest actinic light levels and are displayed from right to left on the x-axis. Values are means ± standard errors (n = 10–15).

The intertidal and subtidal plants in both bay systems exhibited similar qP vs. qN relationships, and qN and qP correlated negatively throughout the experimental period (Fig 4). Although the relationship pattern between qP and qN was similar between the intertidal and subtidal plants, the negative slopes were steeper in the intertidal plants than those in the subtidal plants in both bay systems during all experimental periods (Fig 4). The values of qN in the intertidal plants were always higher than those in the subtidal plants under all light conditions in both bay systems (Fig 4).

### Carbon stable isotope ratio

**Fig 5:**
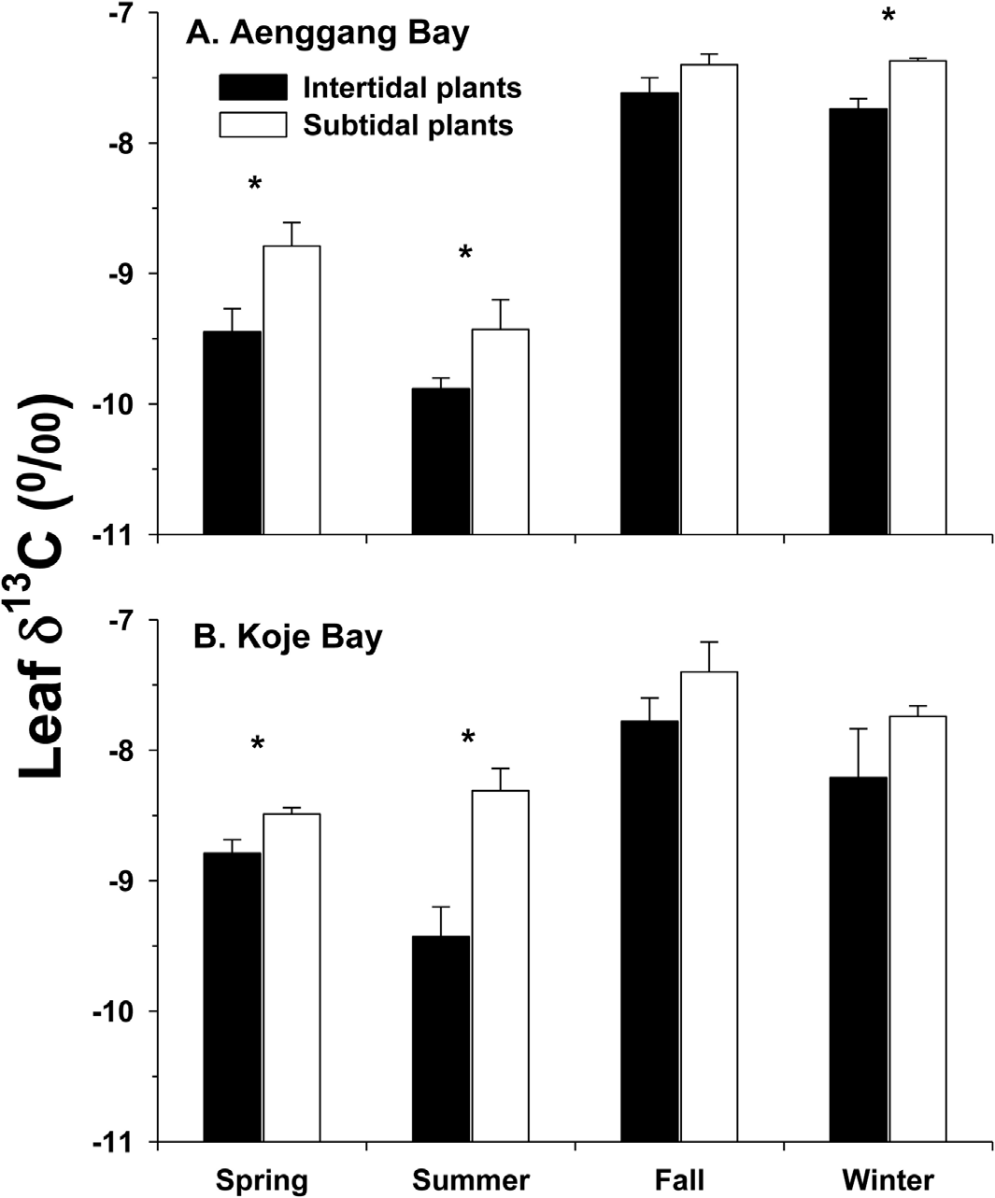
*Zostera marina*. **Carbon stable isotope (δ**^**13**^**C) values of leaf tissues for the intertidal and subtidal plants in Aenggang Bay (A) and Koje Bay (B).** Values are means ± standard errors (n = 6). An asterisk above the bar indicates a significant difference between the intertidal and subtidal plants in each season.

Leaf δ^13^C values were significantly more negative (*P* < 0.01) in the intertidal plants than in the subtidal plants throughout the study period in both bay systems (Fig 5). The differences in the leaf δ^13^C values between intertidal and subtidal plants were more distinguishable during the spring and summer growing season. Leaf δ^13^C values of the intertidal plants in Aenggang Bay ranged from −9.89 to −7.62‰ with a mean of −8.55‰, whereas the values for subtidal plants ranged from −9.43 to −7.37‰ with a mean of −8.38‰ (Fig 5A). Leaf δ^13^C values of the intertidal plants in Koje Bay ranged from −9.43 to −7.78‰ with a mean of −8.58‰, whereas values of subtidal plants ranged from −8.79 to −7.40‰ with a mean of −8.03‰ (Fig 5B).

### Photosynthetic responses to emersion and recovery after re-immersion

**Fig 6:**
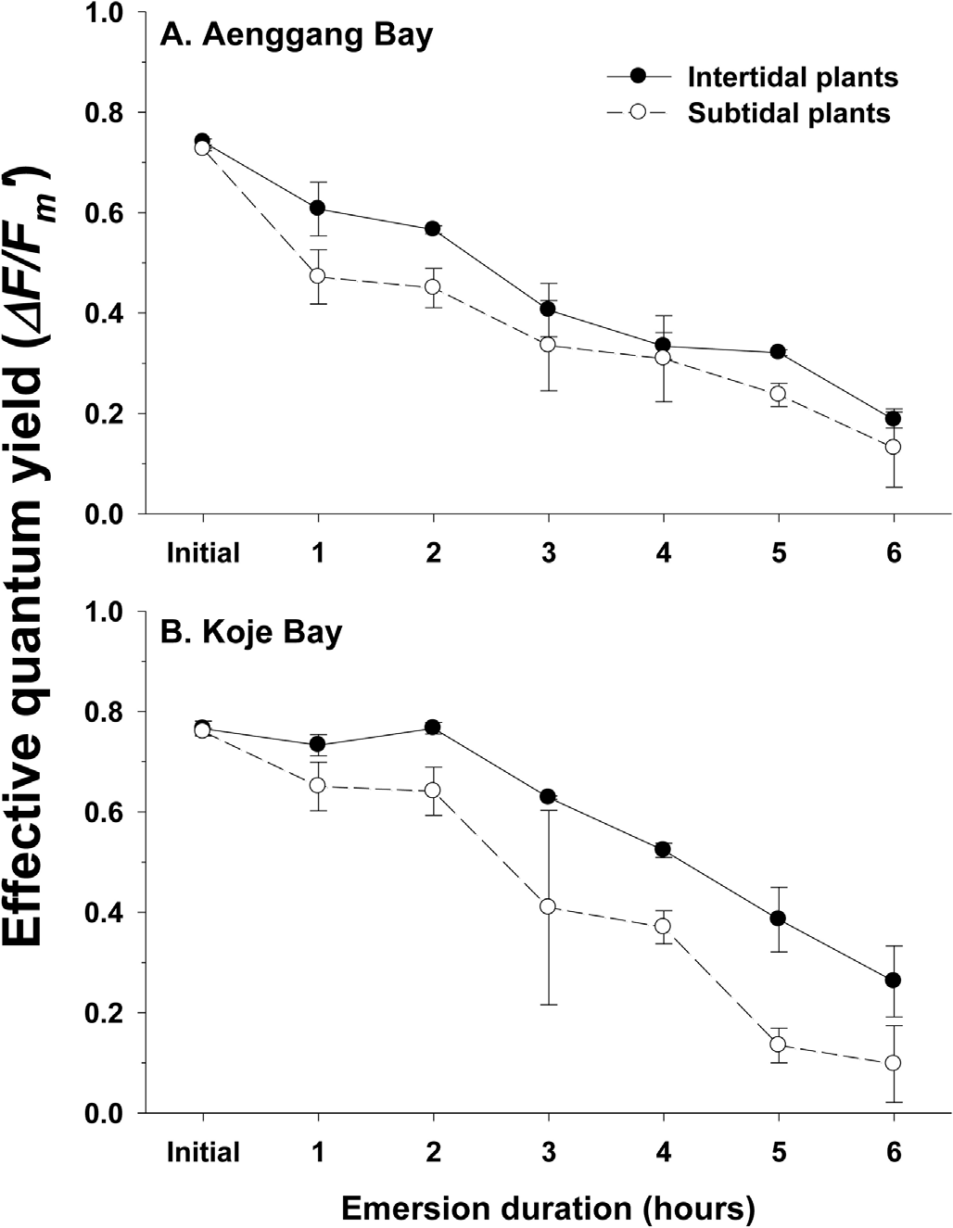
*Zostera marina*. **Effective quantum yields (*ΔF*/*F***_***m***_***´*) in response to emersion time for intertidal and subtidal plants in Aenggang Bay (A) and Koje Bay (B).** Values are mean ± standard errors (n = 10).

Effective quantum yield of the intertidal and subtidal plants decreased gradually with increasing emersion time, and was almost quenched after 6 hours of emersion in both bay systems (Fig 6). After 1 hour of emersion, effective quantum yield of the intertidal plants in Aenggang and Koje Bays decreased by 18% and 4%, respectively, whereas the quantum yield of the subtidal plants in Aenggang Bay and Koje Bay decreased by 35% and 14%, respectively. Effective quantum yield decreased more rapidly by emersion in subtidal plants than in intertidal plants in both bay systems (Fig 6).

**Fig 7:**
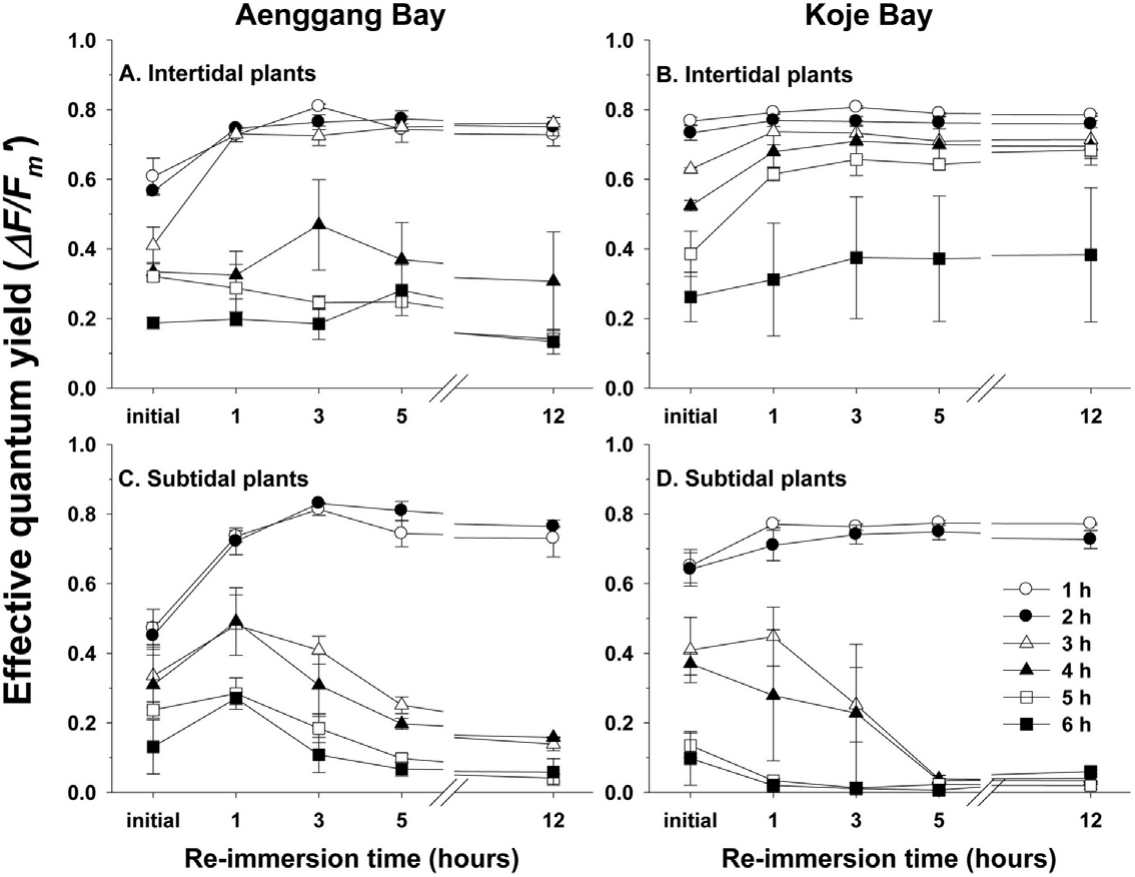
*Zostera marina*. **Recovery of effective quantum yield after 0, 1, 3, 5, and 12 h of re-immersion for the intertidal (A and C) and subtidal plants (B and D) previously exposed to air for 1, 2, 3, 4, 5, and 6 h emersion periods.** Values are means ± standard errors (n = 10).

Photosynthetic recovery from emersion stress exhibited different patterns between the intertidal and subtidal plants as well as among emersion durations (Fig 7). The intertidal plants had greater ability to recover from emersion stress than did the subtidal plants in both bay systems (Fig 7). The intertidal plants in Aenggang Bay, which were exposed to air for 1–3 hours, completely recovered effective quantum yield to the undamaged level after approximately 3 hours of re-immersion, whereas the intertidal plants exposed to air for > 4 hours did not recover effective quantum yield after re-immersion (Fig 7A). The intertidal plants in Koje Bay, which were exposed to air for 1–5 hours, completely recovered effective quantum yield after re-immersion (Fig 7B). Only the subtidal plants in both bay systems, which were exposed to air for < 2 hours, recovered quantum yield after approximately 3 hours of re-immersion (Fig 7C and 7D). The effective quantum yields of the subtidal plants were irreversibly damaged by air exposure for > 2 hours (Fig 7C and 7D).

## Discussion

### Morphology and photosynthetic pigments

Shoot length and leaf width of *Z*. *marina* in the intertidal zone were significantly smaller than those in the subtidal zone. Reduced shoot size in the intertidal zone has been reported for many temperate and tropical seagrasses [10,41,42]. The temperate seagrasses *Z*. *marina* and *Phyllospadix iwatensis* in the intertidal zone have shorter canopy heights and narrower leaf widths than subtidal plants due to desiccation stress during low tide [2,43]. When the plants were transplanted from the subtidal zone to the intertidal zone, leaf sizes of the tropical seagrasses *Cymodocea rotundata* and *Thalassia hemprichii* decreased approaching the sizes of intertidal shoots [10]. The reduction in seagrass shoot size in the intertidal zone has been considered an adaptation for better survival under desiccation stress [9,10]. In the present study, *Z*. *marina* plants in the intertidal zone were regularly exposed to high desiccation stress and excessive light conditions during low tide, which may have resulted in permanent cellular and tissue damages.

The composition of photosynthetic pigments in seagrass leaves varies with light conditions [9,44]. Under reduced light conditions, chlorophyll content in seagrass leaf tissues increases and chlorophyll *a*/*b* ratios decrease [15,16,45]. A higher chlorophyll *a*/*b* ratio has been reported in intertidal *C*. *nodosa* plants, compared to that of subtidal plants [4]. In the present study, total chlorophyll concentrations in subtidal plants were slightly higher than those in intertidal plants, whereas the chlorophyll *a*/*b* ratio was significantly higher in the intertidal plants than in the subtidal plants. Subtidal *Z*. *marina* appeared to adapt to absorb more light under limited light conditions, in contrast to the intertidal plants.

The main role of carotenoids in plants is to protect chlorophyll from photodamage under excess light [46,47]. Dramatic increases in total carotenoid concentrations have been observed in terrestrial and marine plants exposed to high light conditions [48,49]. In the present study, the intertidal plants had higher carotenoid contents than did the subtidal plants during all experimental periods, except winter, in both bay systems. Mean carotenoid concentration of intertidal plants during spring–fall was 1.2 times higher than that of subtidal plants. The intertidal plants were periodically exposed to air during low tide, and thus were probably faced with excess light stress, which increased carotenoid content to avoid damage to the photosystems.

### Photosynthetic characteristics

In the present study, the RLC parameters (rETR_max_, α, E_k_, *F*_*v*_/*F*_*m*_, and NPQ) of *Z*. *marina* varied significantly between intertidal and subtidal plants. The rETR_max_ and E_k_ values of intertidal plants were significantly higher than those of subtidal plants, whereas the α values were higher in subtidal plants than those in intertidal plants. Reductions in rETR_max_ and E_k_ with increasing water depth have been reported in several seagrass species [3,50]. Reduced light availability with increasing water depth was attributed to reduced ETR_max_ and E_k_, and increased α of tropical seagrass species, indicating a photoacclimatory response to low light conditions for more efficient light use [14, 51].

However, rETR_max_, E_k_, and *F*_*v*_/*F*_*m*_ were higher in subtidal plants than in intertidal plants during winter. Dramatic declines in maximum and effective quantum yields at extreme temperatures, which are linked to chronic inhibition of photosynthesis, have been observed in *H*. *ovalis* [52]. Mean air temperature at both bay systems in the present study was approximately 2°C during December–February [53]. Thus, the intertidal plants probably experienced extremely low air temperature when they were exposed to air during low tide in winter. Lower rETR_max_, E_k_, and *F*_*v*_/*F*_*m*_ values in the intertidal plants during winter may have been caused by the low temperatures they encountered during low tide.

Reduced *F*_v_/*F*_m_ under high irradiance has been observed in various marine plants [3,54,55]. Declines in *F*_v_/*F*_m_ under high light fluxes are considered a photoprotective response to PSII photodamage [3,56,57]. Plant leaves adapted to high light environments generally have high photosynthetic capacity and active photoprotective mechanisms [58]. Ralph and Gademann [59] demonstrated that high-light adapted *Z*. *marina* leaves have a high rETR capacity and show active NPQ. In the present study, the intertidal plants had lower *F*_v_/*F*_m_ but higher NPQ than those of the subtidal plants. Additionally, the qP and qN responses of the intertidal and subtidal plants also indicated that the intertidal plants were phenotypically adapted to high ambient light. Thus, the photosynthetic responses exhibited by the intertidal plants were probably associated with photoprotection to minimize photodamage due to excessive irradiance.

### Inorganic photosynthetic carbon source

In the present study, the δ^13^C values of leaf tissues were more negative in the intertidal plants than those in the subtidal plants throughout the experimental period in both bay systems. Seagrasses are adapted to use HCO_3_^−^, which is the most abundant form of dissolved inorganic carbon in seawater, as well as dissolved CO_2_ [60]. As the δ^13^C of seawater HCO_3_^−^ is less negative than those of atmospheric and dissolved CO_2_ [26], marine plants, which can use HCO_3_^−^ for photosynthesis, usually have heavier δ^13^C values, compared to those of terrestrial C_3_ plants [61]. The heavier δ^13^C values in tropical seagrasses are attributed to greater use of HCO_3_^−^ due to a low supply of CO_2_ in seawater [62]. Additionally, lighter δ^13^C values are observed in intertidal *H*. *ovalis* plants than in subtidal plants [63]. The lighter carbon isotope values in intertidal plants have been suggested to be caused by a sufficient CO_2_ supply in seawater due to air mixing during tidal changes [63]. Thus, the lighter carbon isotope values of the intertidal plants in the present study were probably due to direct use of atmospheric or dissolved CO_2_ for photosynthesis during emersion [63–65]. Although the δ^13^C values of intertidal plants were more negative than those of the subtidal plants, intertidal plants in the present study had much heavier carbon isotope values than those of atmospheric and dissolved CO_2_ or terrestrial C_3_ plants [61], suggesting that the intertidal plants in the present study primarily used HCO_3_^−^ in seawater for photosynthesis during immersion.

### Emersion stress and recovery after re-immersion

The desiccation tolerance of intertidal seagrasses has been explained by changes in morphological characteristics and higher shoot density [41,43]. Reduced shoot size, such as shorter canopy height and narrower leaf width in intertidal seagrasses, has been suggested as a morphological adaptation to desiccation stress during low tide [2,9,10,43]. Higher seagrass shoot density in the intertidal zone has also been suggested as an adaptation to reduce desiccation stress through mutual shading of the shoots [43]. In the present study, effective quantum yield of the intertidal plants decreased more slowly than that of the subtidal plants with increasing emersion time, suggesting higher desiccation tolerance in intertidal plants than in subtidal plants. Thus, the intertidal *Z*. *marina* plants in the present study showed photosynthetic adaptation to desiccation stress. The intertidal plants also possessed a greater ability to recover from emersion stress than did the subtidal plants in this study. The intertidal plants that were exposed to air for < 3–5 hours completely recovered photosynthetic ability after re-immersion, but only the subtidal plants that were exposed to air for 1–2 hours recovered photosynthetic ability. This higher desiccation tolerance and recovery ability from photosynthetic damage by intertidal plants may contribute to survival and distribution of this seagrass species in the intertidal zone.

**Fig 8:**
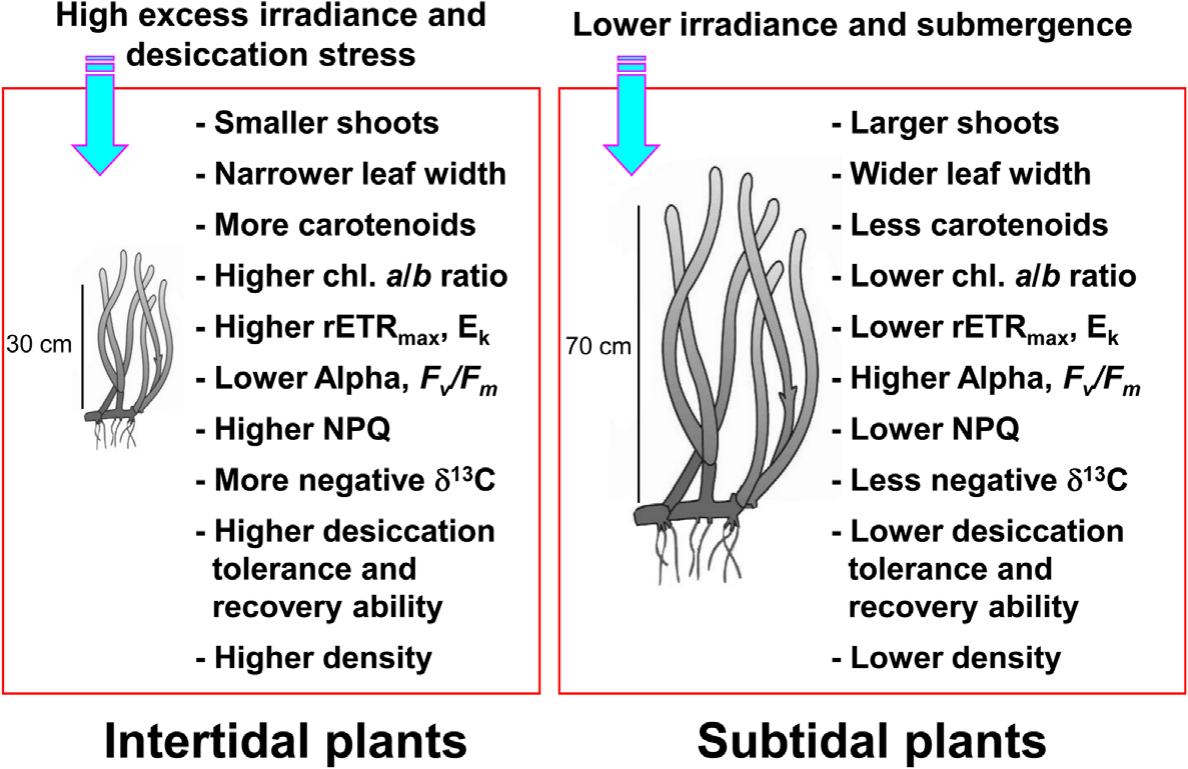
Schematic summary of the morphological and photoacclimatory responses of intertidal and subtidal *Zostera marina* plants.

In conclusion, *Z*. *marina* shoot size, such as shoot height and leaf width, was significantly smaller in plants inhabiting the intertidal zone than in plants inhabiting the subtidal zone (Fig 8). The smaller size of the intertidal plants appeared to be caused by high desiccation stress in the intertidal zone. Lower chlorophyll contents, higher chlorophyll *a*/*b* ratios, and higher carotenoid contents were observed in the intertidal *Z*. *marina* plants than in those in the subtidal zone (Fig 8). We considered the variability in photosynthetic pigment contents between the intertidal and subtidal plants to be a photo-adaptation to effectively use *in situ* light and prevent photodamage. Reductions in rETR_max_ and E_k_ were observed in subtidal plants. However, the α values were higher in subtidal plants than in intertidal plants. Reduced rETR_max_ and E_k_ and increased α in the subtidal plants are suggested photoacclimatory responses to low light conditions (Fig 8). The intertidal plants exhibited higher desiccation tolerance and recovery ability from desiccation damage than did the subtidal plants (Fig 8). The δ^13^C value of leaf tissues was more negative in the intertidal plants than in the subtidal plants, suggesting that the intertidal plants utilized atmospheric or dissolved CO_2_ for photosynthesis during low tide (Fig 8). The morphological and photosynthetic plasticity exhibited by the intertidal and subtidal plants appeared to contribute to the distribution of *Z*. *marina* in both intertidal and subtidal zones. A better understanding of photoacclimatory responses of seagrass plants along the tidal height gradient will promise more accurate prediction of changes in coastal seagrass ecosystems due to global climate associated environmental disturbances, such as a sea level rise.

## Supporting Information

S1 Appendix Photosynthetic pigments, RLC parameters, photochemical quenching (qP), non-photochemical quenching (qN), carbon stable isotope ratios, effective quantum yields in response to emersion time, recovery of effective quantum yield after 0, 1, 3, 5, and 12 h of re-immersion for intertidal and subtidal plants in Aenggang Bay and Koje Bay. Values are mean ± standard errors.
(XLSX)

S1 Table Summary of ANOVA results for morphological characteristics and photosynthetic pigments of *Zostera marina* at the intertidal and subtidal zones in Aenggang Bay and Koje Bay. All data were transformed by log(x+1) to meet the assumption of parametric statistics prior to analysis.
(DOC)

S2 Table Summary of ANOVA results for photosynthetic characteristics of *Zostera marina* at the intertidal and subtidal zones in Aenggang Bay and Koje Bay. All data were transformed by log(x+1) to meet the assumption of parametric statistics prior to analysis.
(DOC)
