# Supplementary material for: Photoacclimatory Responses of Zostera marina in the Intertidal and Subtidal Zones
Source: PLoS One. 2016 May 26;11(5):e0156214. doi: 10.1371/journal.pone.0156214 (PMC4881947; doi:10.1371/journal.pone.0156214)
Supplement: S1 Table — All data were transformed by log(x+1) to meet the assumption of parametric statistics prior to analysis. (DOC) [file pone.0156214.s002.doc]

S1 Table. Summary of ANOVA results for morphological characteristics and photosynthetic pigments of *Zostera marina* at the intertidal and subtidal zones in Aenggang Bay and Koje Bay. All data were transformed by log(x+1) to meet the assumption of parametric statistics prior to analysis

| **Paramter** | **Source** | **df** | **Aenggang Bay** | | |  | **Koje Bay** | | |
| --- | --- | --- | --- | --- | --- | --- | --- | --- | --- |
|  |  |  | **MS** | ***F*-ratio** | ***P*-value** |  | **MS** | ***F*-ratio** | ***P*-value** |
| **Morphological characteristics** | | |  |  |  |  |  |  |  |
| Shoot height | Depth | 1 | 2.586 | 931.984 | < 0.001 |  | 0.875 | 306.125 | < 0.001 |
| ` | Season | 3 | 0.131 | 47.078 | < 0.001 |  | 0.075 | 26.337 | < 0.001 |
|  | Depth × Season | 3 | 0.146 | 52.790 | < 0.001 |  | 0.010 | 3.553 | 0.024 |
|  |  |  |  |  |  |  |  |  |  |
| Sheath length | Depth | 1 | 0.103 | 32.945 | < 0.001 |  | 2.185 | 501.058 | < 0.001 |
|  | Season | 3 | 0.910 | 289.721 | < 0.001 |  | 0.468 | 107.287 | < 0.001 |
|  | Depth × Season | 3 | 1.140 | 362.997 | < 0.001 |  | 0.155 | 35.541 | < 0.001 |
|  |  |  |  |  |  |  |  |  |  |
| Leaf width | Depth | 1 | 1.616 | 1548.837 | < 0.001 |  | 0.598 | 484.233 | < 0.001 |
|  | Season | 3 | 0.365 | 349.691 | < 0.001 |  | 0.068 | 55.033 | < 0.001 |
|  | Depth × Season | 3 | 0.276 | 264.161 | < 0.001 |  | 0.022 | 17.981 | < 0.001 |
| **Photosynthetic pigments** | | |  |  |  |  |  |  |  |
| Total | Depth | 1 | 0.005 | 1.102 | 0.302 |  | 0.014 | 7.028 | 0.013 |
| chlorophyll | Season | 3 | 0.025 | 5.905 | 0.003 |  | 0.017 | 28.807 | < 0.001 |
|  | Depth × Season | 3 | 0.003 | 0.701 | 0.559 |  | 0.065 | 10.711 | < 0.001 |
|  |  |  |  |  |  |  |  |  |  |
| Total | Depth | 1 | 0.012 | 8.524 | 0.007 |  | 0.044 | 64.847 | < 0.001 |
| carotenoids | Season | 3 | 0.016 | 11.210 | < 0.001 |  | 0.015 | 7.539 | 0.001 |
|  | Depth × Season | 3 | 0.001 | 0.477 | 0.701 |  | 0.008 | 3.881 | 0.019 |
|  |  |  |  |  |  |  |  |  |  |
| Chlorophyll | Depth | 1 | 0.016 | 12.908 | 0.001 |  | 0.006 | 8.322 | 0.007 |
| a/b ratio | Season | 3 | 0.014 | 11.274 | < 0.001 |  | 0.484 | 206.619 | < 0.001 |
|  | Depth × Season | 3 | 0.001 | 1.116 | 0.358 |  | 0.009 | 3.993 | 0.017 |
|  |  |  |  |  |  |  |  |  |  |
| Chlorophyll/ | Depth | 1 | 0.013 | 6.917 | 0.013 |  | 0.003 | 6.011 | 0.021 |
| carotenoids | Season | 3 | 0.026 | 14.110 | < 0.001 |  | 0.181 | 142.607 | < 0.001 |
|  | Depth × Season | 3 | 0.001 | 0.190 | 0.902 |  | 0.011 | 8.723 | < 0.001 |
