# Supplementary material for: Photoacclimatory Responses of Zostera marina in the Intertidal and Subtidal Zones
Source: PLoS One. 2016 May 26;11(5):e0156214. doi: 10.1371/journal.pone.0156214 (PMC4881947; doi:10.1371/journal.pone.0156214)
Supplement: S2 Table — All data were transformed by log(x+1) to meet the assumption of parametric statistics prior to analysis. (DOC) [file pone.0156214.s003.doc]

S2 Table. Summary of ANOVA results for photosynthetic characteristics of *Zostera marina* at the intertidal and subtidal zones in Aenggang Bay and Koje Bay. All data were transformed by log(x+1) to meet the assumption of parametric statistics prior to analysis

| **Paramter** | **Source** | **df** | **Aenggang Bay** | | | | | |  | | **Koje Bay** | | | | | |
| --- | --- | --- | --- | --- | --- | --- | --- | --- | --- | --- | --- | --- | --- | --- | --- | --- |
|  |  |  | **MS** | | ***F*-ratio** | | ***P*-value** | |  | | **MS** | | ***F*-ratio** | | ***P*-value** | |
| **Photosynthetic characteristics** | | |  |  | |  | |  | |  | |  | |  | |  |
| rETR_max_ | Depth | 1 | 0.010 | | 2.052 | | 0.159 | |  | | 0.017 | | 1.539 | | 0.218 | |
|  | Season | 3 | 0.0439 | | 87.134 | | < 0.001 | |  | | 0.393 | | 35.731 | | < 0.001 | |
|  | Depth × Season | 3 | 0.023 | | 4.575 | | 0.007 | |  | | 0.042 | | 3.788 | | 0.013 | |
|  |  |  |  | |  | |  | |  | |  | |  | |  | |
| α | Depth | 1 | 0.001 | | 14.088 | | < 0.001 | |  | | 0.002 | | 25.032 | | < 0.001 | |
|  | Season | 3 | 0.004 | | 78.700 | | < 0.001 | |  | | 0.004 | | 49.788 | | < 0.001 | |
|  | Depth × Season | 3 | 0.00008 | | 1.774 | | 0.165 | |  | | 0.00005 | | 0.573 | | 0.634 | |
|  |  |  |  | |  | |  | |  | |  | |  | |  | |
| E_k_ | Depth | 1 | 0.00003 | | 0.095 | | 0.759 | |  | | 0.0001 | | 0.591 | | 0.444 | |
|  | Season | 3 | 0.002 | | 4.811 | | 0.006 | |  | | 0.002 | | 5.290 | | 0.002 | |
|  | Depth × Season | 3 | 0.002 | | 7.652 | | < 0.001 | |  | | 0.002 | | 7.256 | | < 0.001 | |
|  |  |  |  | |  | |  | |  | |  | |  | |  | |
| Effective | Depth | 1 | 0.003 | | 0.201 | | 0.656 | |  | | 0.028 | | 1.955 | | 0.165 | |
| quantum yield | Season | 3 | 0.583 | | 37.314 | | < 0.001 | |  | | 0.669 | | 45.993 | | < 0.001 | |
|  | Depth × Season | 3 | 0.134 | | 8.565 | | < 0.001 | |  | | 0.201 | | 13.792 | | < 0.001 | |
|  |  |  |  | |  | |  | |  | |  | |  | |  | |
| NPQ | Depth | 1 | 0.123 | | 65.780 | | < 0.001 | |  | | 0.135 | | 77.624 | | < 0.001 | |
|  | Season | 3 | 0.024 | | 13.051 | | < 0.001 | |  | | 0.037 | | 20.974 | | < 0.001 | |
|  | Depth × Season | 3 | 0.013 | | 6.963 | | < 0.001 | |  | | 0.006 | | 3.646 | | 0.015 | |
